# Medial Rectus Muscle Injuries after Functional Endoscopic Sinus Surgery

**DOI:** 10.4274/tjo.01328

**Published:** 2015-08-05

**Authors:** Bengi Demirayak, Özgül Altıntaş, Hakan Ağır, Şahin Alagöz

**Affiliations:** 1 Kocaeli University Faculty of Medicine, Department of Ophthalmology, Kocaeli, Turkey; 2 Kocaeli University Faculty of Medicine, Department of Plastic and Reconstructive Surgery, Kocaeli, Turkey

**Keywords:** diplopia, endoscopic sinus surgery, extraocular muscle injury, Strabismus

## Abstract

In recent years, functional endoscopic sinus surgery (FESS) has improved the treatment of sinus disorders. However, various orbital complications have been reported, including optic nerve damage, orbital hemorrhage, infection, lacrimal drainage system injury, and strabismus. Complications are rare but may cause severe morbidity. We describe two patients who underwent endoscopic sinus surgery procedures that resulted in trauma to the medial rectus muscle. The first patient had medial rectus paresia due to contusional trauma and showed spontaneous resolution in a month. The other patient had an orbital medial wall defect with medial rectus injury and he underwent orbitotomy. Medial rectus innervation returned at postoperative 8 months. Several extraocular muscles may be traumatized during FESS. Timing and method of treatment are based on the severity and type of injury and the number of muscles involved. Treatment strategies are dependent on accurate interpretation of magnetic resonance imaging scans.

## INTRODUCTION

The development of transnasal endoscopic sinus surgery (ESS) in recent years presents a greater risk for orbital injury. The close proximity of the paranasal sinuses to the orbita places the orbital contents at risk of injury during sinus surgery, especially surgery of the ethmoid sinuses by a less experienced surgeon.^1,2^ The newer powered devices used for ESS reduce the risk of hemorrhage during sinus surgery but have a greater potential to damage extraocular muscles when accidentally misdirected. Extraocular muscle damage may cause permanent strabismus with troublesome diplopia.

## CASE REPORTS

### Case 1

**Figure 1:**
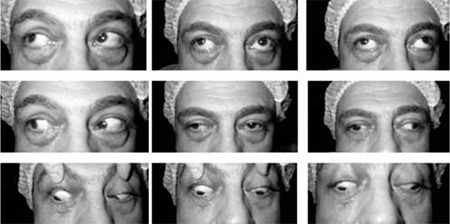
Case 1, 75 PD exotropia in the primary position, increasing to 90 PD on left gaze and decreasing to 14-16 PD on right gaze

A 45-year-old man underwent ESS, during which right orbital hemorrhage and proptosis of the right eye were observed, and lateral canthotomy was performed. The patient was referred with persistent diplopia and face turn to our ophthalmic clinic 8 days after surgery. He was under treatment with 1 mg/kg oral corticosteroid. On examination, visual acuity was normal, and there was no afferent pupillary defect. Adduction was limited about 15 degrees temporal to midline in the right eye. There was 75 PD exotropia in the primary position, increasing to 90 PD in left gaze and decreasing to 14-16 PD in right gaze, with no proptosis (Figure 1). Forced duction test was negative.

**Figure 2:**
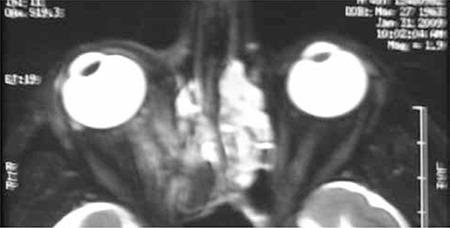
Case 1, MRI revealed bone defect on the right medial orbit wall; the medial rectus was deviated medially and was adherent to this defect. There was no apparent connection between the proximal and distal segments of the muscle

**Figure 3:**
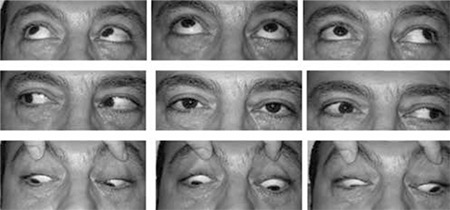
Case 1, at 8 months postoperatively, there was no face turn or exotropia in primary position, and 14 PD exotropia in the left gaze position

Magnetic resonance imaging (MRI) revealed a defect in the medial wall of the right orbit (Figure 2). The medial rectus was deviated medially and was adherent to this defect, although the forced duction test was negative. There was no apparent connection between the proximal and distal segments of the muscle. The patient underwent right orbitotomy. The muscle was found intact, herniated into the bony defect and surrounded by scar tissue. The medial rectus was freed and bony defect repair was performed using an implant. Botulinum toxin (typically 5 units under direct visualization) was injected into the right lateral rectus muscle to weaken it. At 8 months postoperatively innervation returned to the medial rectus muscle. There was no face turn or exotropia in primary position, only 14 PD exotropia in the left gaze position (Figure 3).

### Case 2

**Figure 4:**
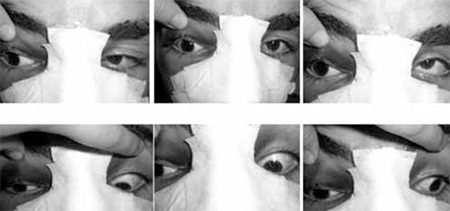
Case 2, adduction was limited about 15 degrees temporal to midline. There was a 45 PD exotropia in the primary position, increasing to 90 PD on left gaze and decreasing to 14 PD on right gaze without proptosis. Forced duction test was negative

**Figure 5:**
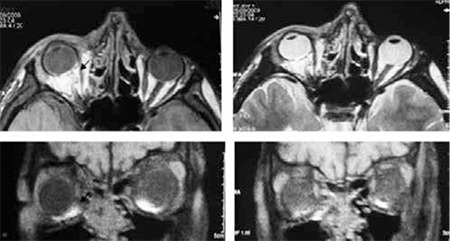
Case 2, MRI revealed enlarged right medial rectus due to hematoma.The medial rectus was paretic due to contusional trauma

**Figure 6:**
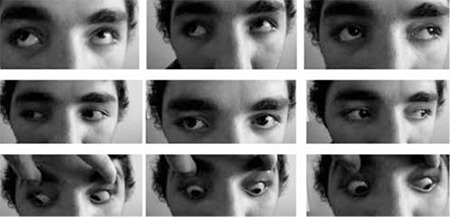
Case 2, Adduction had spontaneously returned to normal after a month.There was still 30 PD exotropia, which was also present in his previous history

A 21-year-old man underwent septoplasty and ESS. During surgery, right orbital hemorrhage, mydriasis and proptosis of the right eye occurred, and right lateral cantothomy was performed as a result. The patient was referred to our ophthalmology clinic on the first postoperative day with diplopia. Visual acuity was 0.5 in the right eye and 1.0 in the left eye. Adduction was limited about 15 degrees temporal to midline. There was 45 PD exotropia in the primary position, increasing to 90 PD in left gaze and decreasing to 14 PD in right gaze, with no proptosis (Figure 4). Forced duction test was negative. MRI revealed an enlarged medial rectus due to hematoma (Figure 5). The medial rectus was paretic due to contusional trauma. Innervation returned to normal spontaneously after only one month. Limitation of adduction disappeared; 30 PD exotropia persisted, which was also present in his previous history (Figure 6).

## DISCUSSION

In recent years, functional ESS (FESS) has become the primary surgical preference for the treatment of medically resistant obstructive sinus disorders. Although a relatively safe procedure, ESS may cause either minor or major complications.^3^ The most common ophthalmic complication is orbital hemorrhage, while other reported orbital complications include optic nerve injury, strabismus and nasolacrimal drainage system injuries.^4^ Ocular motility dysfunction may be seen after ESS due to several mechanisms and can cause strabismus, diplopia and abnormal head positions.^5^ Direct injury to the extraocular muscle is the most common cause of ocular motility complications after ESS.^6,7^ The medial rectus is the most common extraocular muscle injured because of its anatomical location, near the thin and fragile lamina papyracea.^8,9^

When there is suspicion of ocular motility disorder, ocular motility exams and forced duction testing should be performed at the initial exam. To define the location, type and severity of extraocular muscle injury, orbital computed tomography (CT) and MRI are needed. Disruption of the lamina papyracea and bony entrapment of an extraocular muscle is best detected by CT because of the fine osseous detail. Although a bony defect itself may not be directly visualized, an entrapped portion of intraorbital fat or extraocular muscle can be detected using MRI. The bony defect on the medial orbit wall was seen on MRI in case 1 and the medial rectus was adherent to this defect. Despite the incarceration to the bony defect seen on MRI, the forced duction test was negative. This situation was later clarified by intraoperative visualisation of the proximal of the muscle as the adherent segment.

In addition, fat-suppressed coronal T1 and T2 weighted images are particularly helpful in the evaluation of underlying hematomas in the subacute stages, and localized scarring in the chronic stages. The amount of tissue loss is best detected with MRI in the acute to early subacute stages, but in the late stages either MRI or CT should be used. Also, T2-weighted images aid in the diagnosis of edema in an extraocular muscle of normal or almost normal size, as well as in the differentiation of injured muscle from surrounding edema or hemorrhage. These image sequences are very helpful in the delineation of muscle fiber bundles, allowing the diagnosis of transection or laceration.^10^ The added benefit of multi-positional MRI is that it detects muscle contractility.

The management of patients with extraocular muscle injury due to FESS is challenging. Proper treatment requires knowing the mechanism, location and severity of injury. In the early postoperative period, a short trial of systemic corticosteroids may be useful in reducing inflammation and scarring. Clinical examination may determine whether the muscle is palsied and whether a restriction is present, but it should be noted that forced duction may be negative while restriction is present, as we observed in case 1. MRI scans can show muscle integrity. As we have seen in case 2, if the muscle is intact but paretic due to contusional trauma or injury to its nerve, waiting and monitoring clinical improvement should be appropriate. The botulinum toxin may be injected to the antagonist rectus muscle to avoid contracture while waiting for recovery for 3-6 months and sometimes up to 12 months.^11^ In our case, adduction had spontaneously returned to normal after a month.

The optimal timing of surgery has not been definitively determined. If there is no clinical improvement in the first two weeks after injury, exploration and freeing or repair of the medial rectus may be beneficial.^12^ Surgery strategies depend on type and severity of injury. If the muscle has been transected but has a long innervated posterior segment, prompt surgical intervention may allow recovery of this segment.^13^ If the muscle has been transected and a large segment is missing or destroyed, prompt muscle transposition surgery is suggested.^14^ In case 1, we planned surgery early because a bony defect of the medial orbit wall was visible on MRI and the medial rectus was adherent to this defect. During surgery, we found the medial rectus intact but herniated into the bony defect and surrounded by scar tissue.

When the muscle is completely transected, reattachment of the lacerated ends by a hang-back suture to bridge the interposing defect in the muscle can achieve improved primary globe position alignment.^15^ Finding the posterior stump of residual medial rectus deep in the orbit can be challenging. Awad et al.^16^ describe retrieval by means of a subperiosteal medial orbitotomy and Lenart et al.^17^ by a transcutaneous medial orbitotomy through a modified Lynch incision. A transnasal endoscopic approach may offer an alternative for patients in whom a lost medial rectus cannot be recovered by the conventional sub-Tenon approach.^18^

In conclusion, extraocular muscles may be traumatized during FESS. Timing and method of treatment depend on the severity and type of injury and the number of muscles involved. Treatment strategies are dependent on accurate interpretation of clinical examination and CT or MRI scans. Surgical strategy should be evaluated on a case by case basis.
